# Integrative attitudes of Ukrainian war refugees in two neighboring European countries (Poland and Hungary) in connection with posttraumatic stress symptoms and social support

**DOI:** 10.3389/fpubh.2023.1256102

**Published:** 2023-11-16

**Authors:** Judit Kovács, Csilla Csukonyi, Karolina Eszter Kovács, Damian Liszka, Paweł Walawender

**Affiliations:** ^1^Institute of Psychology, University of Debrecen, Debrecen, Hungary; ^2^Institute of Sociology, University of the National Education Commission, Krakow, Poland

**Keywords:** war refugees, Ukraine, mental health, PTSD, economic growth, social support, acculturation attitudes

## Abstract

Since February 24th, 2022, millions of Ukrainians have sought refuge in other, mainly European countries. Hungary, after Poland, is the second largest host of Ukrainian refugees. Only a portion of them are asylum seekers (~11.0% in Poland and ~ 1.1% in Hungary). The aim of the study is to compare the integrative acculturation attitudes between the war refugees residing in the two European countries. The comparison takes into account both the suffering of posttraumatic stress symptoms and social support. It is the first comparative study of this kind pertaining to the Ukrainian refugees in European countries. The data were obtained by a survey method using the modified CAPI (Computer Assisted Personal Interview) technique. The data analyzed were collected between November 21st and December 20th, 2022 from 728 adult Ukrainian individuals who crossed the borders of Poland and Hungary after February 24th, 2022. The research results show that refugees in Poland perceive significantly more social support and show stronger integrative attitudes than refugees in Hungary. The two samples do not differ regarding the presence of posttraumatic stress. The integrative attitudes proved not to be linked to gender and age, but linked to the host country. Besides social support and the host country, posttraumatic stress also proved to be a significant predictor of integrative attitudes.

## 1. Introduction

According to Maughan and Burgess, there are two main aspects of social sustainability relating to mental health. The first is the ability to rebuild the social capital lost due to the mental illness (like employment, education, or housing) and the second is the required societal support (including the support provided by health care professionals, organizations, and wider public) (1).

Humanitarian crises, such as those caused by natural disasters or wars, are associated with an increased prevalence of mental disorders (like depression and PTSD) as violence is a major threat to mental health (2).

Millions of Ukrainians have sought refuge in other, mainly European countries since February, 24th, 2022. According to the UNHCR statistics, by September 6th, 2023 there were 6.201.600 Ukrainian war refugees recorded worldwide (3) resulting in the worst migration crisis in Europe since World War II (4). The migration was involuntary. Refugees had been forced to move in order to maintain personal safety. They did not migrate for better economic or educational opportunities (5).

Exposure to armed conflict has been associated with negative mental health consequences. Hoppen and Morina estimated in their meta-analysis that 354 million adult war survivors suffer from posttraumatic stress disorder (PTSD) and major depression (because of wars which occurred between 1989 and 2015) (6). Individuals with PTSD suffer from the persistent recollections of the traumatic events and experience the avoidance of stimuli. They are associated with the trauma accompanied by general unresponsiveness and increased arousal, irritability, sleep disorders, and impaired attention (7).

The Russo-Ukrainian war began at a time when the Ukraine population was already in a poor state of physical and mental health because of the Covid-19 pandemic. An armed conflict occurring immediately after a pandemic creates an atmosphere of uncertainty and instability that negatively influences mental health, especially for those who are directly affected by the war (8). Additionally, the healthcare system of Ukraine was compromised even during pre-pandemic times. It was challenged badly during the pandemic. Now, it is suffering severely mainly by the damaged infrastructure due to the war (9).

Studies examining the connection between the war and the mental health of Ukrainians report that people experience worsening mental health after the Russia’s attack in February 24th, 2022 (10–13). Most notable are increases in the incidence of anxiety, depression and loneliness (11). High rates of PTSD have also been documented in war-affected populations (12). In their study, Kuparov and colleagues found that Ukrainians that stayed in Ukraine reported less symptoms of anxiety, depression and stress than those who moved abroad (13).

One of the earliest published articles dealing with the mental health of the Ukrainian war refugees was a study based on a sample of 304 individuals surveyed in Germany. It showed that severe psychological distress, severe depressive symptoms and anxiety are prevalent for a significant portion of refugees (40.5 and 20.4%, respectively) (14). In line with this observation, another study also reported that Ukrainian refugees staying in Poland showed severe depressive and anxiety symptoms at a comparable level (~30% of the 623 individuals) (15).

The question arises whether posttraumatic stress urges individuals to adapt to the new culture, or, on the contrary, makes the successful adaptation more difficult. On one hand, we can expect that traumatic memories make returning to the home country impossible (an urge to adapt to the current environment). On the other hand, severe symptoms may make taking initiatives more difficult.

The literature is inconclusive regarding the relationship between posttraumatic stress and acculturation. Stasielowicz (16) studied the adaptive performance of refugees who experienced trauma living in Germany. Adaptive performance refers to “altering behavior to meet the demands of a new situation, event, or set of circumstances” (17, p. 615). It can be needed at a workplace, at any new institution, or system in the host country. The author found that posttraumatic stress was negatively related to task-oriented adaptive performance.

Nonetheless, there are studies which found a positive, or no relationship between posttraumatic stress and acculturation. One of them sought to examine whether PTSD or major depression inhibits psychosocial adaptation in refugees. In the research participation in and dropouts from a short integration-supporting program held in Australia for refugees from the former Yugoslavian countries was monitored. The study found that neither PTSD nor depression were related to dropouts (18). Jurcik et al. (19) also reported that acculturation and PTSD are not related when examining the relationship in a sample of refugees residing in Canada.

However, a study examining the relationship between trauma, acculturation, and mental health symptoms in Somali refugees in the United States (20) resulted in quite an unexpected finding. Various acculturation dimensions (American cultural identity, English language competency, and American cultural competency) occurred despite traumatic experiences and mental health symptoms.

Thus, sometimes posttraumatic stress may be an inhibiting factor, sometimes a neutral one, and sometimes a facilitating factor in regards to cultural adaptation. We propose that social support can be related to this relationship. If an individual receives the necessary help, then posttraumatic stress can facilitate participating in the life of the host country.

According to the UNHCR data, only a portion of the refugees in Poland and Hungary are asylum seekers. In Poland, by September 2023, out of 14.881.470 Ukrainian refugees that crossed the Polish border, 1.639.725 were asylum seekers (~11.0%). Meanwhile in Hungary, only 37.565 individuals were asylum seekers out of the 3406.310 that entered Hungary (~1.1%) (21). These two ratios are obviously very different. Hungary, after Poland, is the second largest recipient of Ukrainian refugees. Nonetheless, it is not the country of destination. Ukrainians usually stay only a few days and move onward to Western European countries (4, 22).

On one hand, governmental policies, resources, services of the host country, community attitudes toward immigrants are crucial to the settlement of refugees. On the other hand, empowerment and participation of the asylum seekers in local communities (23) are also key to success. Applying for asylum in a certain country is a sign of an acculturative purpose with respect to that country. Immigrants find their way in a host society in various ways. We can distinguish between assimilation, integration, separation, and marginalization in the framework of the well-known cultural adaptation strategies theory of Berry (24). Assimilation means adapting to the culture of a host country and putting aside the migrant’s home culture. Separation involves keeping the original culture and rejecting the host country one. Integration is the maintaining of the original culture while including compatible aspects of the culture of the new country of stay. Lastly, marginalization is not adapting to the new culture and losing the original one (5, 25). The framework incorporates two dimensions: contact with members of the origin culture and contact with members of the host culture. Integration (contact with members of both the origin and the host culture) is desirable from the perspective of the host culture. It can also be beneficial for refugees’ mental health.

Integration supporting initiatives, like, e.g., simplified path to employment, were implemented in both countries. Nonetheless, two aspects of key importance in influencing the mental state and acculturation of immigrants are very different in the countries. First, Poland has been one of the favorite destinations of Ukrainian immigrants well before the war started. The contemporary migrations from Ukraine to Poland started in the 1990s. Ukrainians became the largest migrant group in Poland (26) and Poland was the main European Union country which Ukrainians choose for work (27). Consequently, war-refugees could find substantial social support in Poland from other individuals from the Ukrainian culture. Social support can mitigate refugees’ isolation and loneliness by enhancing the sense of belonging, and facilitate integration into a new society (28–30). Additionally, language competencies crucially influence the success of cultural adaptation (31).

Social support refers to the support available through interpersonal connections (32). Weinert and Brandt (33) conceptualized social support mainly in the nursing context. Their concept was based on the five underlying factors identified by Weiss (34): the sense of being valued, being an integral part of a group, perceived chances for intimacy, perceived opportunities for nurturance, perceived availability of information, emotional and material help. The measuring tool from this approach, the PRQ-2000 (35), has been successfully implemented in studies dealing with cultural adaptation with respect either to migrant people (36) or international students (37). The tool has showed consistently that acculturation stress is lower when social support is stronger.

Social support appears to be a very important element linked to cultural adaptation irrespective of the specific method used for data collection. An interview-study involving service providers and policymakers in health and immigrant settlement in Canada showed that social support is perceived to play an important role in immigrant settlement and to have a positive impact on immigrant health (29). Social support is a substantial factor not only from the perspective of service providers and policymakers, but immigrants themselves emphasize its role. Liamputton and Kurban studied young Middle-Eastern refugees living in Melbourne with a qualitative approach addressing how they perceive their wellbeing and the barriers they face in their new homeland. The researchers identified the presence or absence of social support (from family, teachers, church, etc.) as a key factor influencing the refugees’ ability to adapt to their new environment (28). Questionnaire based studies, other than those using the PRQ-2000, also showed that social support perceived by refugees is linked with positive mental state characteristics. In a study of minority Syrian refugees living in Iraq, Yildirim and colleagues (38) found social support (measured by the Multidimensional Scale of Perceived Social Support) (39) to be related with flourishing. The flourishing embraced several forms of personal resources (engagement with daily activities, being respected by others, having a purposeful life, and being optimistic about the future) (40).

The aim of this study is to compare the integrative acculturation attitudes of war refugees residing in Poland and Hungary. Posttraumatic stress symptoms are taken into account in addition to social support. The mental state of refugees can also be connected to acculturation in addition to the structural-environmental factors (like language difficulties, the presence of others from the home culture who can provide social support, national legislation, etc.). This study is both interdisciplinary and international. It is the first comparative study of this kind taking into account the Ukrainian war refugees in European countries.

Our findings may also have practical implications. The results may provide guidance for policymakers planning actions toward Ukrainian refugees in European countries. This may prevent some of the refugees who are able and willing to work from falling into long-term unemployment, especially the not in employment, education or training (“NEET”) status. Reducing long-term unemployment and the number of people in the “NEET” status has been the highest priority in the European Union youth employment policy (41).

We expected refugees surveyed in Poland to show a stronger integrative attitude than those surveyed in Hungary (H1). Simply put, this expectation was based on the statistics regarding the number of asylum-seekers in each country.

We also expected refugees from Poland to report a stronger integrative attitude even after controlling for social support (H2).

Finally, we investigated, without formulating explicit expectations, the potential link between social support and the relationship between posttraumatic stress and integrative cultural adaptation.

## 2. Materials and methods

### 2.1. Method

The data were collected in face-to-face interviews through a survey method using the modified CAPI (Computer Assisted Personal Interview) technique. The study used a standardized measurement tool - a questionnaire also containing psychological scales enabling the characterization of the respondents in terms of their emotional state. This was important due to the traumatic circumstances of the migration. The sample was selected according to a scheme known as “random-route.” In this selection method, the pollster receives a selected address called the “starting point.” Next, the pollster starts the research conducting successive interviews in every nth apartment, starting from the starting point.

The survey was carried out among Ukrainians aged 18 and over who crossed the Polish-Ukrainian and Hungarian-Ukrainian borders after the date of Russia’s armed invasion (February 24, 2022). The structure of the defined population was not known. The pollsters selected the respondents in accordance with the sample instructions and gave the respondents a mobile device on which the survey tool was uploaded. The respondents completed the questionnaire by themselves. The data was automatically recorded in the system and ready for analysis, so there was no need for manual recording. Automation accelerated the processing of the results.

### 2.2. Sample

The size of the population of Ukrainian refugees residing in Poland and Hungary was estimated in September, 2022 based on the latest UNHCR data available: 1.017.655 individuals in Poland (the number of active registrations in the PESEL database, from around the 1.5 million refugees who applied for a temporary protection; those who left the country for over 30 days were excluded) and 28.662 individuals in Hungary (the number of applications for temporary protection). The total estimated population size was approximately 1.05 million Ukrainians. According to the outlines in the table by Krejcie and Morgan (42), a sample size of 384 is suggested as appropriate for a population of one million. The data collection was conducted during one month period (from November 21st until December 20th, 2022) in Poland and Hungary. In total, 807 valid surveys were obtained. Due to the dynamic size and structure of the population, it was assumed that in order to achieve an appropriate selection of the sample the population should be divided into strata from which individuals were then drawn for the sample. Our research reached respondents from all the Ukrainian oblasts, including the occupied Autonomous Republic of Crimea, Luhansk, and Donetsk oblasts. The selection criteria were as follows: (1) War refugee – the respondent first entered the territory of Hungary or Poland after the date of Russia’s invasion of Ukraine (February 24th, 2022); (2) Ukrainian resident - the respondent was a resident of Ukraine before first entering the territory of Poland or Hungary; (3) Adult – the respondent was at least 18 years old; (4) Not a Hungarian minority – the minority was excluded from the data.

For the purpose of this study, we used data obtained from 728 Ukrainians (see Table 1).

**Table 1:** Demo-social characteristics of the studied sample.

Variables		Poland, Valid *N* = 400	Hungary, Valid *N* = 328	Differences
*Age*	Mean (SD) Min-Max	M = 36.8 (SD = 12.2) 18–65	M = 37.8 (SD = 10.9) 18–63	*p* = 0.180
*Gender* % (*N*)	Female	90% (359)	71% (233)	*p* = 0.0000
	Male	10% (41)	29% (95)	
*Place of residence in Ukraine* % (*N*)	Village	14% (57)	17% (56)	*p* = 0.0000
	Small town (up to 20 thousand inhabitants)	16% (62)	20% (67)	
	Medium city (from 20 to 99 thousand inhabitants)	23% (92)	32% (106)	
	Large city (from 100 to 500 thousand inhabitants)	26% (104)	16% (51)	
	Very large city (over 500 thousand inhabitants)	21% (85)	15% (48)	

The respondents arrived from all 24 Ukrainian oblasts, as well as one autonomous republic and two cities with a special status (Autonomous Republic of Crimea, Donetsk Oblast, and Kherson Oblast, respectively) (see Supplementary Table S1).

The distribution of oblasts that Ukrainian refugees arrived from likewise differed between Poland and Hungary, *p* < α (*p* = 0.0000). Residents whose oblast capitals are very large and large cities of Ukraine (Kyiv, Lviv, Donetsk, Odessa, Kherson) relocated to Poland more often. Refugees in Hungary were mainly residents of Transcarpathia, Kyiv Oblast, and oblasts close to the border with Hungary (Ivano-Frankivsk).

### 2.3. Measurements

Personal Resource Questionnaire (PRQ 2000) (35). The 15-question PRQ 2000 is designed to measure the perceived level of social support. It is graded on a 7-point Likert scale that corresponds to the level of agreement (from 1 = “strongly disagree” to 7 = “strongly agree”). The score range is from 15 to 105, where a higher score denotes a higher level of perceived social support. Sample items are: “There is someone I feel close to who makes me feel secure” or “I belong to a group in which I feel important.” The scale had a good consistency in our study (α = 0.942).

The Posttraumatic Stress Disorder Checklist-5 (PCL-5) (43). This is a 20-item self-report instrument, which assesses symptoms of PTSD as defined by the DSM-5. The 20 items of the PCL-5 reflect the frequency with which respondents have experienced the item in question rated on a 5-point Likert-scale ranging from “not at all” to “extremely.” A total score (0–80) can be obtained by summing up the scores for each of the 20 items. A clinical diagnosis cannot be achieved based purely on the score obtained from the PCL-5. However, scores at or above the cut-off score of 33 are indicative of provisional PTSD. Sample items include: “Feeling very upset when something reminded me of the stressful experience.” or “Avoiding memories, thoughts, or feelings related to the stressful experience.” The Ukrainian version of the PCL-5 questionnaire used on a large sample (*N* = 2,203) in 2016 showed a high reliability of the scale (α = 0.960) (44), just like in our study (α = 0.944). Even the subscales of PCL-5, except the two-items Avoidance (α = 0.564), showed sufficient consistency (Re-experiencing: α = 0.821; Negative alterations in cognitions and mood: α = 0.857; Alterations in arousal and reactivity: α = 0.856).

Integrative cultural adaptation attitudes (45). These attitudes were measured using some of the items measuring integrative attitudes of the Acculturation Attitudes Scale (AAS). The AAS is a 56-item instrument that assesses 14 areas of individual and family life. Out of the fourteen areas we used society (“While living in Poland / Hungary, I would like to retain the Ukrainian cultural heritage and lifestyle and participate fully in various aspects of life of the Polish / Hungarian society”), values (“I would encourage children to educate and to participate fully in various aspects of life of Polish / Hungarian society, while teaching them Ukrainian values and customs”), success (“To be successful, we must participate fully in various aspects of life of the Polish / Hungarian society, but also maintain our Ukrainian culture and heritage”), and interests (“Ukrainians should join together and form organizations to represent Ukrainian interests in Poland / Hungary, and also actively participate in Polish / Hungarian organizations”). The consistency of the answers for the four items translated into Ukrainian for the purpose of this study was sufficient (α = 0.762). We used the average score of the agreement with the statements on the 5-points scale as an index for the integrative attitude.

### 2.4. Research model

Linear regression analysis was used for hypothesis testing. Three models were built: The first one incorporates age, gender and host country as the main demographic variables and examines how these variables are related to integrative attitudes. The second model adds the psychological variables of social support and posttraumatic stress, as independent variables. Adding psychological variables to demographic ones makes it possible to detect the potential change in the relationship between demographic variables and integrative attitudes. Particularly, we expected the connection between the country and the integrative attitude to decrease, since we anticipated social support to be the primary explanation for the relationship between country and integration. Lastly, the third model included the interactive component of social support × posttraumatic stress.

## 3. Results

Table 2 shows the scores of the distinguished variables in the samples from the two host countries. The consistency of measurements proved to be high with respect to all three variables (αs > 0.75).

**Table 2:** Social support, posttraumatic stress and integrative attitudes (M/SD) in the two samples (PL, HU).

Variables	Hungary	Poland	Differences
Social support	67.707 (18.901)	77.122 (14.411)	Z = −7.129 *p* = 0.0000
Posttraumatic stress	28.863 (15.368)	26.355 (16.942)	Z = −1.736 *p* = 0.083
Integrative attitude	4.380 (1.206)	4.994 (0.993)	Z = −7.067 *p* = 0.001

We can see that refugees in Poland perceive significantly more social support and show stronger integrative attitudes than refugees in Hungary. The two samples do not differ regarding the presence of posttraumatic stress symptoms. In Hungary, 45.9% of females and 44.2% of males fall into the group experiencing provisional PTSD (scored more than 32 points) (45.4% of the whole sample), while in Poland it was 45.7% of females and 36.6% of males (44.8% of the whole sample).

The integrative attitudes proved not be linked to gender and age. Nonetheless, they were linked to the host country (β = 0.263) (see Table 3). The coefficient of the host country decreases after incorporating social support into the model (β = 0.131) since the host country and social support are correlated, see the first row of Table 2, but, it remains significant. Thus, Poland remains attractive beyond the greater amount of perceived social support. The effect size of social support proved to be large (β = 0.572). Besides social support and the host country, posttraumatic stress is also a significant factor with a positive coefficient in the second model, with a small effect size (β = 0.161). The coefficient remains positive and significant (β = 0.142) even after incorporating the interactive component (social support × posttraumatic stress) in the third model. This interactive component also proved to be a significant predictor of integrative attitudes, with a very small effect size (β = 0.067) (see Table 3).

## 4. Discussion

Presented study investigated the integrative cultural adaptation strategy of Ukrainian war refugees in relation to social support and posttraumatic stress in two countries, Poland and Hungary. The refugees’ appearance and cultural practices are mostly similar to those dominant in Poland and Hungary. Both countries are neighboring countries. Both are very receptive and helpful towards Ukrainian refugees, and both have supportive legislation encouraging employment. Yet, a much larger portion of refugees are asylum-seekers in Poland than in Hungary. The difference between the two countries can be explained to some extent by social support considering the large number of Ukrainians present in Poland even before the war. Moreover, Poland may be also more attractive to Ukrainians even besides social support due to the other factors, such as the similarity between the spoken languages.

**Table 3:** Integrative attitude in connection with demographic variables, social support and posttraumatic stress (standardized β coefficients are listed).

Model No.	Variables	1	2	3
1.	*Age*	0.021 *p* = 0.556	0.050 *p* = 0.094	0.045 *p* = 0.135
	*Gender (1 = female, 2 = male)*	−0.029 *p* = 0.426	0.011 *p* = 0.724	0.010 *p* = 0.738
	*Country (1 = Poland, 0 = Hungary)*	0.263 *p* = 0.0000	0.131 *p* = 0.0000	0.132 *p* = 0.0000
2.	*Social support*		0.572 *p* = 0.0000	0.589 *p* = 0.0000
	*Posttraumatic stress*		0.161 *p* = 0.0000	0.142 *p* = 0.0000
3.	*Social support × posttraumatic stress*			0.067 *p* = 0.042
	*R^2^ Significance in R^2^ change*	0.074 *p* = 0.0000	0.372 *p* = 0.0000	0.375 *p* = 0.042

Data from Ukrainian war-refugees were collected at the end of 2022 in Poland and in Hungary about integrative attitudes (45), social support (35), and posttraumatic stress (43).

The distribution of age and gender of the respondents differed between Poland and Hungary. The average age of the respondents in Hungary was slightly higher than of respondents in Poland However, this is not a statistically significant result, *p* > α (*p* = 0.180). There were also differences in gender between the host countries, *p* < α (*p* = 0.0000). The surveyed population was very female biased. A greater degree of female bias was visible among refugees in Poland, where women accounted for 90% of the sample. In the Hungarian sample women comprised 71%.

There were also differences in the characteristics of the place of residence of the Ukrainian refugees. Most of the respondents from Hungary lived in medium-sized and small towns before first arriving in the host country. The largest number of respondents from Poland lived in very large and large cities in Ukraine.

A substantial part of the refugees (approx. 44% in both countries) were found to have a severe level of posttraumatic stress (PCL-5 scores >32).

We hypothesized and found that the integrative attitude is more positive in Poland (H1). It remained more positive even after controlling for social support (H2). Perceived social support proved to be the strongest correlate of integrative attitudes. This is in agreement with what others found with different methods in different studies (28, 29, 38). We expected perceived social support to be related to the relationship between posttraumatic stress and the integrative adaptation purposes. What we found, a weak but significant relation, is in line with the common sense of social support being most needed in severe life situations in order to overcome the crisis. The positive relationship between posttraumatic stress and integrative attitudes remained even after controlling for this interaction.

The high level of provisional PTSD prevalence is above the level that meta-analyses report with respect to war refugees (approx. 30%) (46, 47). This high ratio of provisional PTSD emphasizes the urgency for help to be provided by professional services, even if perhaps the majority of refugees would recover without professional intervention. Similarly to Rousseau and colleagues (48) findings with respect to war-refugees residing in Canada.

We believe that the separate effect of the host country (stronger integrative purpose in Poland) came, at least in some part, from the closeness of the spoken languages, Polish and Ukrainian. The differences between the meanings of some words can be small when distinguishing between two languages with the same origin (49). It unquestionably helps immigrants to find jobs, cope with everyday challenges, and communicate with members of the host country (50–52). Gaining language proficiency has a significant impact on the well-being of immigrants (53). Hungarian is a language “island” in Europe surrounded by Neo-Latin, Germanic, and Slavic languages (54). Polish is a West Slavonic Language, while Ukrainian and Russian languages (spoken by 97% of Ukrainians), are East Slavonic Languages. Both originated from Common Slavic, also called the Proto-Slavic language (55). Polish, Russian, and Ukrainian spoken languages overlap substantially. In Hungary, even English cannot function as a mediating language, since millions of Hungarians do not speak English at all (56). In short, language competence is a key factor in acculturation and mental health (31, 50–52). Nonetheless, in the presented study we did not measured the host country language proficiency among the respondents. Thus, this is an area to be explored in some future research.

We can corroborate the consensual knowledge in literature that social relationships are needed for social integration (e.g., (28, 29, 38)). On a very general level, the concept of the need to belong (57) explains why an individual may lose adjustment to society without possessing satisfactory social relationships. The survey addressed not the support received from the host society in general, but mainly support from close relationships. Perceived social support proved to be the strongest correlate of integrative attitudes. It would be useful to distinguish whom the help comes from to get a clearer picture of the role that close relationships play in forming integrative attitudes. We found that the relationship between the host country and integration decreased when perceived social support was added to the model. This can be explained on the basis of many Ukrainians residing in Poland even before the war. Among the limitations of our study, there is a lack of detailed information on the relationships of the refugees with culturally experienced Ukrainians that have been living in the host country since the time before the war.

After reviewing the literature, where controversial results can be found (16, 18–20), we introduced perceived social support into the system of relationships to investigate the integrative component of posttraumatic stress and social support and its relation with cultural adaptation. Although the interaction mattered and a positive relationship of posttraumatic stress with integrative attitudes was stronger when individual perceives being socially supported, the separate positive link of posttraumatic stress with integration did not disappeared from the model. This is in line with the findings of Jorgenson and Nilsson (20) with respect to Somali refugees in the United States. They found better English language and American cultural competencies among refugees with more traumatic experiences and more mental health symptoms.

The concept of posttraumatic growth has the potential to make a bridge between controversial findings. Posttraumatic growth, as Tedeschi and Calhoun write “is the experience of positive change that occurs as a result of the struggle with highly challenging life crises. It is manifested in a variety of ways, including an increased appreciation for life in general, more meaningful interpersonal relationships, an increased sense of personal strength, changed priorities, and a richer existential and spiritual life” (58). Posttraumatic stress disorders and posttraumatic growth can coexist. PTSD can positively influence posttraumatic growth among refugees as Von Arcosy and colleagues (59) found. Having data not only about posttraumatic stress, but also about posttraumatic growth could offer a clearer picture about the connection between trauma and cultural adaptation in a new country.

Several limitations should be acknowledged in this study, including the ones concerning the sample features such as incomplete data about the population of Ukrainian war refugees in both countries. The challenge was in preparing an exhaustive sampling frame of refugees. For example, in Poland refugees registered in the PESEL database, which enabled them to obtain social benefits. Nonetheless, the database lacked some records, like a record of the place of residence. Another limitation about the sample features was related to the population dynamics. The fluctuation of refugees is understandable given the situation from which they are fleeing and the opportunities they will seek in other countries. For researchers, this is a challenge in terms of difficulties in estimating and interpreting the results. Moreover, there were no data obtained about the time the sample population had spent in Poland and in Hungary. We mentioned that Hungary remains a transient country for most of the Ukrainian refugees who stay there for a few days. This was stated based only on reference to external data. Such a comparison requires further investigation which is beyond the scope of our study. Another limitation is that we do not have direct information from our respondents on their language competence in the language of the host country. We also do not have specific information with respect to the origin of the received social support. We should add that we do not have individual information on war experiences or how the respondents were affected by the war, whereas literature data clearly shows that mental health consequences of war are strongly connected to how one is affected by the war (12). Moreover, the distribution of the home oblasts was different in the two samples. This implicates potentially different reasons for moving west; however, we have not addressed the motivation for migrating in our questionnaire. Instead, we supposed homogenous motives of searching for a safer life. Obviously, in a large-sample questionnaire study the very complex phenomenon of being a war refugee cannot be fully studied.

A practical conclusion of the study results is that early actions consisting of providing mental health support, social support, and communicative language competences undertaken by policymakers in European countries accepting war refugees from Ukraine can change the integrative attitudes of the refugees. Moreover, they can reduce the risks of long-term unemployment and its resource consequences for health care systems (60) and rebuild the two main aspects of social sustainability relating to mental health - the social capital lost due to the mental illness and to the societal support. Adult women comprised 90% of the Polish and 71% of the Hungarian sample (at the end of 2022). Therefore, European policymakers should consider various scenarios while supporting the integration process of the refugees. Employment may not be the best solution for women of all ages. Moreover, integration with the labor market should consist of various tools and procedures to support the Work-Life-Balance of working adults, especially that many of the refugees have dependents or are older people (61–63).

## Data availability statement

The raw data supporting the conclusions of this article will be made available by the authors, without undue reservation.

## Ethics statement

The studies involving humans were approved by Institutional Ethics Committee of University of Krakow Rector’s Research Ethics Board (protocol code DNa.0046.1.5.2022, 28.09.2022) and Egyesített Pszichológiai Kutatásetikai Bizottság (protocol code 2022-108, 08.10.2022). The studies were conducted in accordance with the local legislation and institutional requirements. The participants provided their written informed consent to participate in this study.

## Author contributions

JK: Conceptualization, Formal analysis, Investigation, Methodology, Resources, Software, Validation, Visualization, Writing – original draft, Writing – review & editing. CC: Conceptualization, Formal analysis, Investigation, Methodology, Resources, Software, Validation, Visualization, Writing – original draft, Writing – review & editing. KK: Conceptualization, Formal analysis, Investigation, Methodology, Resources, Software, Validation, Visualization, Writing – original draft, Writing – review & editing. DL: Conceptualization, Funding acquisition, Investigation, Project administration, Resources, Supervision, Visualization, Writing – original draft, Writing – review & editing. PW: Conceptualization, Funding acquisition, Investigation, Project administration, Resources, Supervision, Visualization, Writing – original draft, Writing – review & editing.
