# Supplementary material for: Integrative attitudes of Ukrainian war refugees in two neighboring European countries (Poland and Hungary) in connection with posttraumatic stress symptoms and social support
Source: Front Public Health. 2023 Nov 16;11:1256102. doi: 10.3389/fpubh.2023.1256102 (PMC10687397; doi:10.3389/fpubh.2023.1256102)
Supplement: Supplementary file 1 [file Table_1.DOCX]

Supplementary Material

Integrative attitudes of Ukrainian war refugees in two neighboring European countries (Poland and Hungary) in connection with post-traumatic stress symptoms and social support

**Judit Kovács, Csilla Csukonyi, Karolina Eszter Kovács, Damian Liszka*, and Paweł Walawender**

*** Correspondence:** Corresponding Author: damian.liszka@up.krakow.pl

# Supplementary Table

**Table S1**. The Ukrainian oblast in which respondents lived before first crossing the Hungarian/Polish border.

| **Odblast** |  | **Poland** | **Hungary** |
| --- | --- | --- | --- |
| *Donetsk* | N | 27 | 19 |
|  | % | 6,8% | 5,8% |
| *Dnipropetrovsk* | N | 27 | 12 |
|  | % | 6,8% | 3,7% |
| *Kyiv City* | N | 53 | 36 |
|  | % | 13,3% | 11,0% |
| *Kharkiv* | N | 44 | 32 |
|  | % | 11,0% | 9,8% |
| *Lviv* | N | 53 | 5 |
|  | % | 13,3% | 1,5% |
| *Odessa* | N | 23 | 14 |
|  | % | 5,8% | 4,3% |
| *Luhansk* | N | 5 | 9 |
|  | % | 1,3% | 2,7% |
| *Autonomous Republic of Crimea* | N | 1 | 15 |
|  | % | 0,3% | 4,6% |
| *Zaporizhzhia* | N | 29 | 20 |
|  | % | 7,2% | 6,1% |
| *Kyiv* | N | 33 | 32 |
|  | % | 8,3% | 9,8% |
| *Vinnytsia* | N | 9 | 14 |
|  | % | 2,3% | 4,3% |
| *Poltava* | N | 9 | 15 |
|  | % | 2,3% | 4,6% |
| *Ivano-Frankivsk* | N | 7 | 11 |
|  | % | 1,8% | 3,4% |
| *Khmelnytskyi* | N | 11 | 11 |
|  | % | 2,8% | 3,4% |
| *Cherkasy* | N | 3 | 13 |
|  | % | 0,8% | 4,0% |
| *Zhytomyr* | N | 9 | 11 |
|  | % | 2,3% | 3,4% |
| *Zakarpattia* | N | 4 | 12 |
|  | % | 1,0% | 3,7% |
| *Mykolaiv* | N | 8 | 11 |
|  | % | 2,0% | 3,4% |
| *Sumy* | N | 2 | 7 |
|  | % | 0,5% | 2,1% |
| *Rivne* | N | 4 | 8 |
|  | % | 1,0% | 2,4% |
| *Chernihiv* | N | 6 | 4 |
|  | % | 1,5% | 1,2% |
| *Kherson* | N | 14 | 3 |
|  | % | 3,5% | 0,9% |
| *Ternopil* | N | 7 | 2 |
|  | % | 1,8% | 0,6% |
| *Volyn* | N | 6 | 4 |
|  | % | 1,5% | 1,2% |
| *Kirovohrad* | N | 4 | 2 |
|  | % | 1,0% | 0,6% |
| *Chernivtsi* | N | 2 | 3 |
|  | % | 0,5% | 0,9% |
| *Sevastopol City* | N | 0 | 3 |
|  | % | 0,0% | 0,9% |
| **Total** | **N** | **400** | **328** |
|  | **%** | **100,0%** | **100,0%** |
